# Methods for Extracellular Vesicles Isolation in a Hospital Setting

**DOI:** 10.3389/fimmu.2015.00050

**Published:** 2015-02-13

**Authors:** Matías Sáenz-Cuesta, Ander Arbelaiz, Amaia Oregi, Haritz Irizar, Iñaki Osorio-Querejeta, Maider Muñoz-Culla, Jesus M. Banales, Juan M. Falcón-Pérez, Javier Olascoaga, David Otaegui

**Affiliations:** ^1^Multiple Sclerosis Unit, Neuroscience Area, Biodonostia Health Research Institute, San Sebastián, Spain; ^2^Spanish Network on Multiple Sclerosis, Madrid, Spain; ^3^Department of Liver and Gastrointestinal Diseases, Biodonostia Health Research Institute, Donostia University Hospital, San Sebastián, Spain; ^4^University of the Basque Country, San Sebastián, Spain; ^5^National Institute for the Study of Liver and Gastrointestinal Diseases (CIBERehd, Instituto de Salud Carlos III), Madrid, Spain; ^6^Ikerbasque - Basque Foundation for Science, Bilbao, Spain; ^7^Asociación Española Contra el Cáncer, Madrid, Spain; ^8^Metabolomics Unit, CIC bioGUNE, Derio, Spain; ^9^Department of Neurology, Donostia University Hospital, San Sebastián, Spain

**Keywords:** extracellular vesicles, protocol standardization, clinical application, nanoparticle tracking analysis, flow cytometry, translational research, urine

## Abstract

The research in extracellular vesicles (EVs) has been rising during the last decade. However, there is no clear consensus on the most accurate protocol to isolate and analyze them. Besides, most of the current protocols are difficult to implement in a hospital setting due to being very time-consuming or to requirements of specific infrastructure. Thus, our aim is to compare five different protocols (comprising two different medium-speed differential centrifugation protocols; commercially polymeric precipitation – exoquick – acid precipitation; and ultracentrifugation) for blood and urine samples to determine the most suitable one for the isolation of EVs. Nanoparticle tracking analysis, flow cytometry, western blot (WB), electronic microscopy, and spectrophotometry were used to characterize basic aspects of EVs such as concentration, size distribution, cell-origin and transmembrane markers, and RNA concentration. The highest EV concentrations were obtained using the exoquick protocol, followed by both differential centrifugation protocols, while the ultracentrifugation and acid-precipitation protocols yielded considerably lower EV concentrations. The five protocols isolated EVs of similar characteristics regarding markers and RNA concentration; however, standard protocol recovered only small EVs. EV isolated with exoquick presented difficult to be analyzed with WB. The RNA concentrations obtained from urine-derived EVs were similar to those obtained from blood-derived ones, despite the urine EV concentration being 10–20 times lower. We consider that a medium-speed differential centrifugation could be suitable to be applied in a hospital setting as it requires the simplest infrastructure and recovers higher concentration of EV than standard protocol. A workflow from sampling to characterization of EVs is proposed.

## Introduction

Extracellular vesicles (EVs) are membrane-bound particles shed from almost all cell types, carrying components from the cell donor such as lipids, proteins, RNA, glycolipids, and metabolites (1). It has been suggested that they play several biological roles like, for example, antigen presentation without cell contact (2), microenvironment modification, and distant cell education (3), roles that have been encompassed under the term “cell-to-cell contact-free communication”. In turn, their biological functions have been related to many pathophysiological processes, the most studied being cancer (4), immune-mediated diseases (5), and cardiovascular disorders (6).

A widespread concern in the biomedical research community is the gap between the basic research carried out in the laboratories and the clinical setting where the new biological information should have a direct impact. Many researchers have directed their efforts toward bridging that gap and look for ways to translate lab findings into clinical solutions, emerging therefore the translational research. The translational research on EVs is not foreign to this goal: the current knowledge about EVs, mostly developed *in vitro*, has been proposed to be applied in a daily hospital routine giving answers to specific health queries (7–13). This possible application ranges from diagnostic to therapeutic objectives, including disease monitoring and the search of prognostic biomarkers, among others. But are the hospitals technologically prepared to employ EVs studies routinely?

The main steps for studying EVs and applying the results involve sampling (blood, urine, saliva, cerebrospinal fluid, joint fluid, breast milk, ascitic fluid, etc.) and isolation, to be afterward characterized and analyzed their cargo and, finally, give a potential clinical interpretation and application. Concerning the first steps, sampling and pre-analytical parameters have been widely studied and are close to reach a consensus (14, 15). However, isolation is still a critical step due to several reasons. First, the methods to isolate EVs are currently highly diverse [reviewed by Momen-Heravi et al. (16) and Witwer et al. (17)] and depending on which one is employed, the results can be considerably different, even having started from the same sample. At the moment, most of them are based on EV density, including differential centrifugation steps from low speeds (1,500 × *g*) to ultracentrifugation (>100,000 × *g*), combined or not with density gradient and/or filtration. Precipitation using polymers and immunoaffinity agglutination are also widely used. Recently, the size-exclusion chromatography (18) and chip devices (19) have been added to the rest of methods. All of them, either by themselves or in combination, yield a solution enriched in EVs in different extents. Finally, depending on several factors such as time consumption, cost, friendly use, and reproducibility, these methods are or not able to be applied in a daily clinical routine. Despite lots of important works shedding light on this field, there is still a lack of consensus (20) evidencing the urgent need of standardized protocols appropriate for hospitals.

Considering the problems and needs regarding the use of EVs in a clinical setting, we established the following objectives for the present study: to compare several protocols for EVs isolation and to analyze which of them could be the most suitable one to be used in daily clinical setting.

## Material and Methods

Blood and urine are the most widely used samples in a hospital setting, as they provide useful information and they are easy to obtain with minimally invasive techniques. Thus, we decided to isolate EVs from these biofluids as the starting point for EV isolation.

Samples were collected from 10 healthy individuals (5 males and 5 females; average age = 37 ± 8 years old) and stored according to the criteria of the Donostia node of the Basque Biobank. All subjects gave written informed consent and the study was approved by the Hospital Ethics Committee.

Donors underwent a questionnaire about recent exercising (within the last hour), drugs/medication intake, ovulatory cycle, acute illness, and sleeping hours.

**Figure 1:**
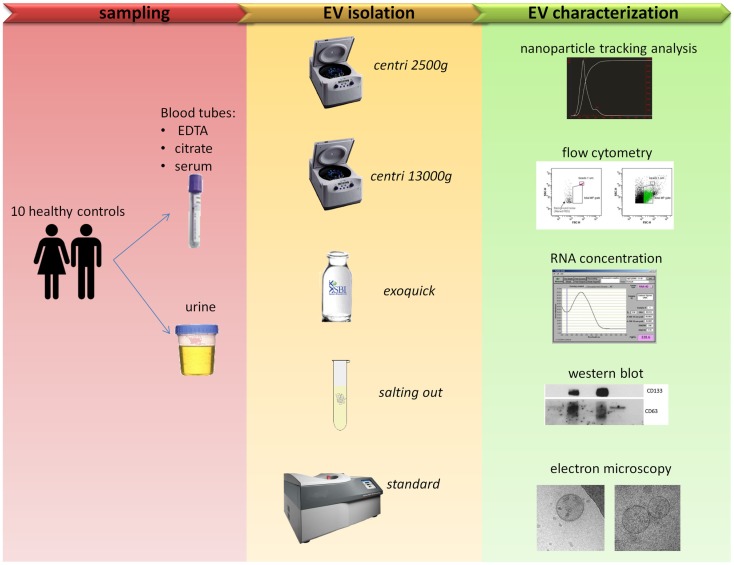
**Comparison of five protocols for EVs isolation**. Blood and urine samples were isolated with five different protocols (see Table 1 for more details) and then characterized with five outputs methods.

The workflow followed in the present work is summarized in Figure 1.

**Table 1:** **Lab parameters analyzed in blood and urine samples**.

Blood	Creatinine (mg/dl)	0.9 ± 0.3
	Total cholesterol (mg/dl)	184.9 ± 37.7
	HDL (mg/dl)	69.9 ± 14.1
	Triglycerides (mg/dl)	65.7 ± 20.1
	LDL (mg/dl)	101.9 ± 37.5
	Total proteins (g/dl)	7.2 ± 0.6
	Albumin (g/dl)	4.3 ± 0.3
	Hematocrit (%)	41.7 ± 3.4
	Leukocyte (10e3/μl)	7.0 ± 2.5
	Platelet count (10e3/μl)	238.7 ± 56.7
	Lymphocyte count (10e3/μl)	1.9 ± 0.5
Urine	Density (g/l)	1019.4 ± 7.7
	pH	6.0 ± 0.9
	Glomerular filtrate (mL/min/1.73 m^2^)	84.3 ± 15.5
	Erythrocyte count (ery./μl)	Negative
	Leukocyte count (leu./μl)	Negative
	Epithelial cell count (cells/μl)	Negative

*Values represent the mean ± SD from the 10 healthy controls*.

### Blood

Peripheral blood samples were collected at the Donostia University Hospital at 8:30 a.m. on fasting and were processed separately (without pooling them) within the first hour. After discarding the first milliliter, blood collection was done by venipuncture with a 21-gage needle in a 10-ml EDTA tube, a 3.8-ml citrate tube, and a 8-ml serum tube [Vacutainer, Becton Dickinson (BD)], kept upright and centrifuged at 2,500 × *g* during 15 min. The supernatant was recovered to obtain platelet-poor plasma (PPP) or platelet-poor serum, being these samples the starting point for all the protocols. Besides, additional blood samples were collected in EDTA and serum tubes to perform a hemogram and obtain protein and lipid profiles in the core laboratory of the Hospital. The parameters tested are shown in Table 1.

Although we collected plasma and serum from peripheral blood, the present work is focused on plasma. In this sense, we are going to refer only to plasma in all the sections of “Materials and Methods” for the sake of simplicity. The results and discussion of blood-derived EVs will be also centered on plasma and only relevant results will be presented in the case of serum.

### Urine

Sixty milliliter-first void of the day was collected in aseptic conditions by each individual at home, kept at 4°C until their processing with an average time of 2.7 h between collection and processing. Ten milliliters were sent to the core laboratory for the analysis of the most common urine parameters (Table 1). The rest was aliquoted in five tubes of 10 ml and centrifuged at 2,500 × *g* for 15 min in order to obtain cell-free urine (CFU).

#### EV isolation protocols

The protocols described below are summarized in Table 2.

**Table 2:** **EV isolation methods compared in this work**.

Method	Isolation principle	Steps
*Centri2500*	Differential centrifugation	2500 × *g* 15′ × 2 + 20,000 × *g* 20′ to pellet the EVs
*Centri13000*	Differential centrifugation	2500 × *g* 15′ + 13,000 × *g* 2′ + 20,000 × *g* 20′ to pellet the EVs
*Exoquick*	Agglutination–precipitation	2500 × *g* 15′ + agglutination with exoquick + 1500 × *g* 30′ and 5′ to pellet the EVs
*Salting out*	Precipitation	2500 × *g* 15′ + 13000 × *g* 30′ + acid precipitation + 5000 × *g* 10′ to pellet the EVs
*Standard*	Differential centrifugation – size filtration – ultracentrifugation	2500 × *g* 15′ + 0.22-μm filter + 10,000 × *g* 30′ + 1,00,000 × *g* 75′ to pellet the EVs

##### Centri2500

This method is based on the protocol published by Lacroix and colleagues (14). Briefly, 1.3 ml of PPP or 9.5 ml of CFU obtained at the first centrifugation are centrifuged again at 2,500 × *g* during 15 min to get 1 ml of platelet-free plasma (PFP) or 9 ml of debris-free urine (DFU). Both PFP and DFU samples were stored at −80°C for later use. When needed, samples were thawed on ice and centrifuged once again at 20,000 × *g* during 20 min to pellet the EVs, discarding 900 μl of supernatant, following the protocols described by Ashcroft and colleagues (21) and Jayachandran and colleagues (22). The pellet containing the EVs was resuspended in 100 μl of PBS (GIBCO, Life Technologies) filtered twice through a 0.22 μm-pore filter.

##### Centri13000

This method is based on the protocol published by Dey-Hazra and colleagues (23) and Dignat-George and colleagues (24). In brief, it is a modification of the previous method where the second centrifugation performed on the 1.3 ml of PPP was done at 13,000 during 2 min to obtain PFP or DFU. The rest of the protocol was the same as the previous one. To note, this protocol was not performed for urine samples.

##### Exoquick

The basis for this method lays on the precipitation of EVs using a commercial agglutinating agent and was performed following the manufacturer’s instructions. In summary, 63 μl or 2 ml of exoquick TC (System Biosciences) were added either to 250 μl of PPP or to 9.5 ml of CFU, respectively, and the mix was incubated overnight at 4°C with no rotation. Then, two centrifugation steps were performed at 1,500 × *g* for 30 and 5 min, respectively, to sediment the EVs and the pellet was resuspended in 200 μl of PBS. It needs to be noted that, although the first versions of the manufacturer’s instructions included a filtering step using a 0.45 μm-pore filter, it was removed in the latest version and, thus, it has not been included in our protocol.

##### Salting out

This method has been adapted from the protocol recently published by Brownlee and colleagues (25) and it is based on the precipitation of EVs through an aggregate of sodium acetate 1 M, ph 4.75. A centrifugation was performed on 1.3 ml of PPP or 9.5 ml of DFU at 13,000 × *g* for 30 min; we collected the supernatant (1 ml of PPP or 9 ml of DFU), added sodium acetate (dilution 1/10), and incubated on ice for 60 min and, subsequently at 37°C for 5 min. The dilution was then centrifuged at 5,000 × *g* during 10 min and the resulting pellet was washed with a buffer with sodium acetate at 0.1 M to be finally resuspended in 200 μl of PBS.

##### Standard

This is the method considered as the standard isolation protocol nowadays (26). The starting point was 1.3 ml of PPP or 9.5 ml of CFU that were filtered through a 0.22 μm-pore filter and centrifuged at 10,000 × *g* during 30 min to obtain either PFP or DFU. These were ultracentrifuged at 100,000 × *g* in an Optima MAX tabletop centrifuge (Beckman Coulter) during 75 min. The resulting EV pellet was resuspended in 200 μl of filtered PBS.

#### EV detection and characterization methods

##### Nanoparticle tracking analysis

The size distribution and concentration of EVs were measured using a NanoSight LM10 machine (NanoSight). All the parameters of the analysis were set at the same values for all samples and 1 min-long videos were recorded in all cases. Background was measured by testing filtered PBS, which revealed no signal. The EVs obtained from PFP (5 μl) were diluted with filtered PBS to 1:150 and the ones obtained from DFU (5 μl) to 1:50. For each sample, two measurements were performed. It is necessary for a minimum of 200 tracks (movements of single particles recorded by a camera) to obtain valid results. The following parameters were measured: the mean and mode of the size distribution and the concentration of EVs (27).

##### Flow cytometry

The labeling and gating of EVs were performed as described by Sáenz-Cuesta and colleagues (28). Briefly, 4 μl of CD61-PE (Cytgonos) or CD45-PE (BD) monoclonal antibodies were mixed with 40 μl of resuspended EVs and incubated for 20 min. Next, labeled EVs were washed once with 300 μl of filtered PBS, resuspended in further 200 μl of filtered PBS and acquired at low rate in a FACS Canto II flow cytometer (BD). Side and forward scatter were measured on a logarithmic scale with the threshold set at 300 for each parameter to avoid instrument noise (background signal). Then, the lower limit was defined with the exclusion of background noise given by the signal of PBS filtered twice. To define the upper limit of the total MP gate, 1-μm non-labeled polystyrene latex beads were used (Sigma-Aldrich). The events that appeared in this region were included in the total EV count and were further analyzed for specific labeling (positive for PE marker). We defined CD61+ EVs as platelet-derived EVs (PEV) and CD45+ EVs as leukocyte-derived EVs (LEV). The total and cellular origin-specific EV concentrations were obtained using Trucount™ tubes [BD; Ref. (28)].

##### Western blot

Primary CD133 (Miltenyi Biotec S. L) and CD63 (Santa Cruz Biotechnology, Inc.) antibodies were used to study specific EV transmembrane markers. Mouse and rabbit HRP-conjugated antibodies (Cell Signaling) were employed as secondary antibodies. All protein procedures were done at non-reducing conditions. Samples (10 μl of PBS-resuspended EVs) were incubated at 95°C for 5 min, separated in SDS polyacrilamide gels, and transferred to nitrocellulose membranes (GE Healthcare). Membranes were blocked for 1 h at room temperature with 5% milk (w/v) in TBS solution with 0.1% Tween-20 (T-TBS) and incubated in the same solution with primary antibodies overnight at 4°C. Primary antibodies were washed with the T-TBS solution and incubation with secondary HRP-conjugated antibodies was performed at room temperature for 1 h in the same solution used for the primary antibodies. After washing with T-TBS solution, the HRP signal was detected by a chemiluminiscent reaction with the SuperSignal West Dura Extended Duration Substrate (Thermo Fisher Scientific, Inc.).

##### RNA isolation

A 185 μl-aliquot of resuspended EVs was used to extract total RNA with the miRNeasy serum/plasma kit (Qiagen). RNA concentration was measured with the nanodrop 1000 spectrophotometer (Thermo Scientific).

##### Cryo-electron microscopy

The cryo-electron microscopy (EM) was performed following the protocol used by Perez and colleagues (29). Briefly, 10 μl of EV preparations were directly adsorbed onto glow-discharged holey carbon grids (QUANTIFOIL Micro Tools GmbH). Grids were blotted at 95% of humidity and rapidly plunged into liquid ethane with the aid of VITROBOT (Maastricht Instruments B). Vitrified samples were imaged at liquid nitrogen temperature using a JEM-2200FS/CR transmission cryo-electron microscope (JEOL) equipped with a field emission gun and operated at an acceleration voltage of 200 kV.

#### Statistical analysis

The statistical analysis was performed with PASW Statistics v18.0 (SPSS Inc.). Kolmogorov–Smirnov and Shapiro–Wilk tests were used to check normality of distributions. As all of the variables were shown to follow a normal distribution, *T*-tests were applied to assess differences between the groups. Pearson’s *R* correlations were computed to explore the relations between lab parameters and some EV parameters. Both differences between groups and correlations between variables were considered significant when *p* < 0.05.

## Results

### Blood

#### EV concentration

Extracellular vesicles concentration was measured using two independent methods: nanoparticle tracking analysis (NTA) and conventional flow cytometry (FC). It is to be noted that the lower detection limits are different, being 50 nm for NTA (27) and around 400 nm for FC (30).

#### Nanoparticle tracking analysis

**Figure 2:**
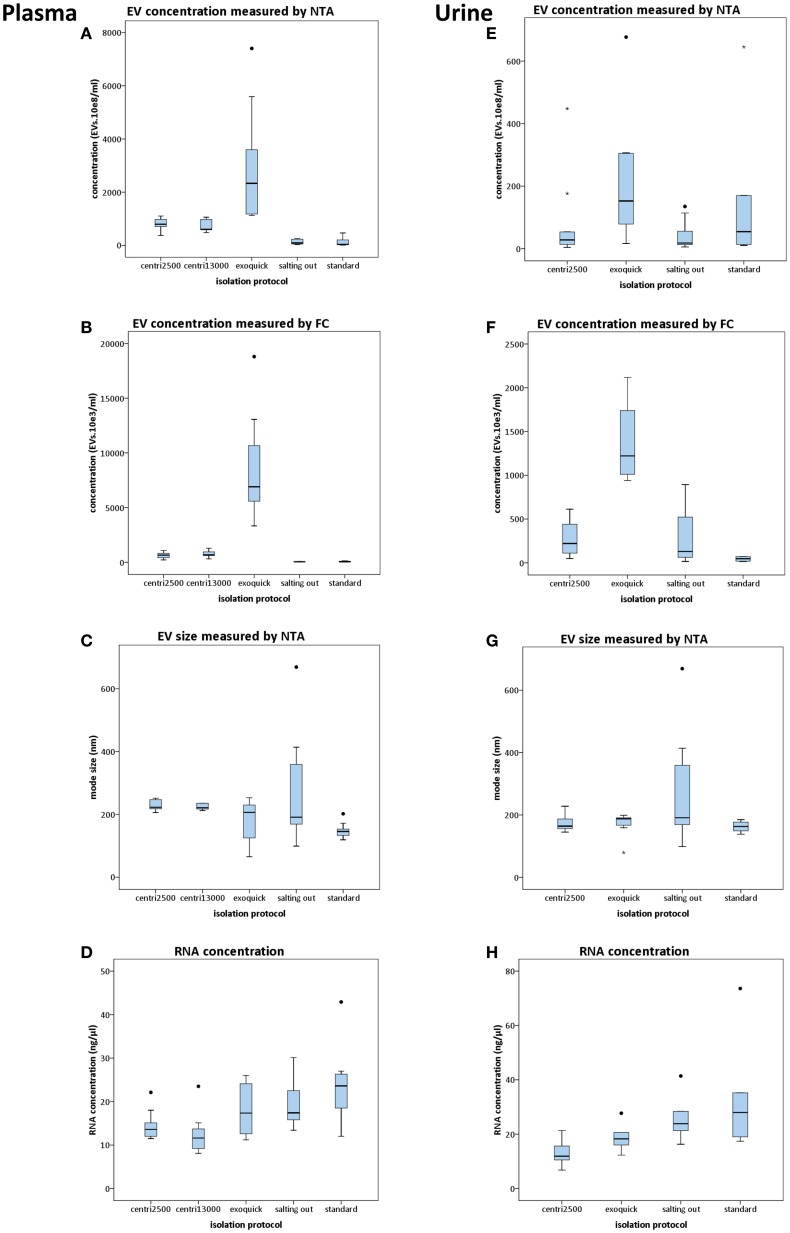
**Results of the comparison of five protocols for isolation of EVs**. Box plots show EV concentration measured by nanoparticle tracking analysis [NTA; **(A,E)**] or conventional flow cytometry [FC; **(B,F)**], EV size distribution measured by NTA **(C,G)**, and concentration of RNA yielded from EVs **(D,H)**. In the left column, the results from plasma-derived EVs are shown and in the right column those from urine. For statistical significance, see text. All bars represent mean values with SD except for size plots **(C,G)** that bars indicate mode with SD.

When using NTA, the software requires a minimum of 200 tracks during the capture time of the video. In the case of the samples processed with the salting out and standard methods, only few of them reached that minimum. This was critical for NTA analysis causing a high variability on these samples (Figures 2A,E). The exoquick method yielded higher EV concentration values than any other method used. We obtained four times higher EV concentration with exoquick than with centri2500 (*p* = 0.007) and centri13000 (*p* = 0.05) and 23 times higher concentration values comparing to salting out (*p* = 0.002) and standard (*p* = 0.002) methods (Figure 2A). No significant differences have been found either between the EV concentrations obtained with the centri2500 and centri13000 methods, or between those yielded by the standard and the salting out methods. However, there are significant differences between these two groups of methods (Figure 2A) obtaining a *p* value of <0.001 for both centri2500 vs. salting out and standard and 0.002 and <0.001 for centri13000 vs. salting out and standard respectively. Regarding the EVs isolated from serum, they were obtained using the exoquick, centri13000, and standard protocols and we have observed that, as it happens with plasma, the exoquick method yields significantly higher EV concentrations than the other two methods. When we compared serum and plasma considering all isolation methods, 3.4 times higher EV concentrations have been observed for serum using exoquick and 1.3 times higher ones when using centri13000, but these comparison did not reach statistical significance (see Table S1 in Supplementary Material).

##### Flow cytometry

Although the EV concentrations obtained using FC were lower than those obtained by NTA, there was good correlation between the concentration profiles yielded by each approach when using the averages for each isolation method for the comparison (*R* = 0.99; *p* < 0.001). Nevertheless, no significant correlations were observed when performing the analysis for each isolation method separately. Besides, significant differences were observed between all isolation methods except for *centri2500* vs. *centri13000* and *salting out* vs. *standard* (Figure 2B).

#### EV size

**Figure 3:**
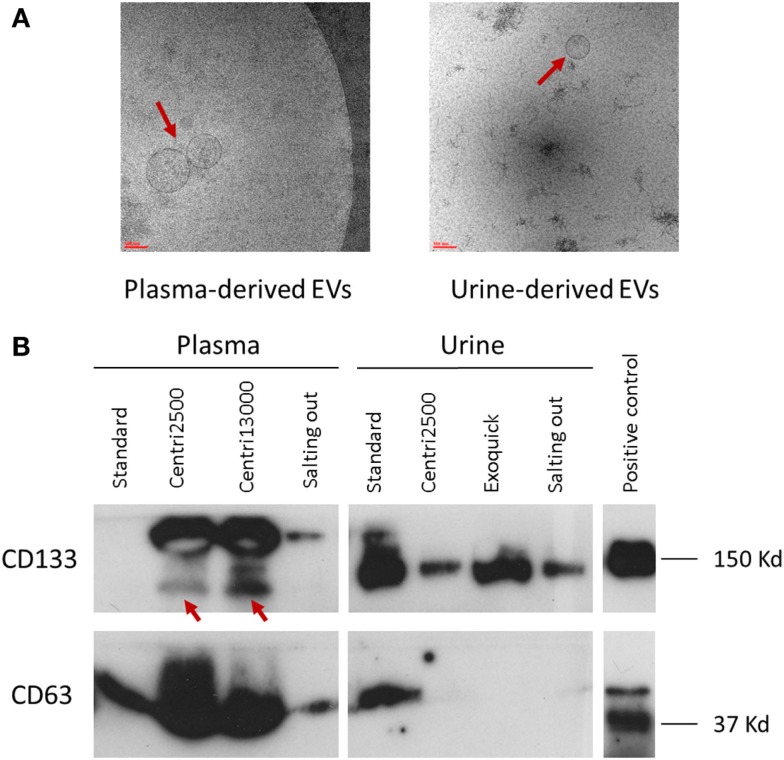
**Characterization of EVs**. **(A)** Two images of electronic microscopy of EVs (pointed with red arrows) derived from plasma and urine. Scale bar represents 100 nm. To note, in the image of urine-derived EVs, it is observed contaminants not seen in the plasma-derived one. **(B)** Western blot analysis using the specific EV markers CD133 and CD63. In CD133 plasma samples, the specific bands are pointed with red arrows. Positive control was performed with NHC-2 p10 cell line.

There is great similarity between the modes of EV size obtained with the different isolation protocols ranging from 150 to 277 nm, average of mode size: 228 nm (Figure 2C). In accordance to these results, the EM images show EVs with a size between 100 and 200 nm (Figure 3A). Significant differences in size only exist between the EVs isolated with the *standard* method when compared to those obtained through the *centri2500* and *centri13000* methods, being the former the smallest of all at 158.7 nm. Besides, the EM images of the EVs obtained with *exoquick* present several filamentous aggregates and other globular structures not considered EVs.

#### RNA concentration in EVs

Despite the *salting out* and the *standard* methods being the ones that yield the lowest EV concentrations, the highest RNA concentrations have been obtained through these methods (19.5 ± 5.7 and 23.6 ± 8.2 ng/μl, respectively), higher than with the *centri2500* and *centri13000* methods (14.5 ± 3.3 and 12.5 ± 4.5 ng/μl, respectively). It is remarkable that *exoquick* yields 18.1 ± 6.0 ng/μl of RNA, despite having isolated around 23 times more EVs than the *salting out* and *standard* methods (Figure 2D).

#### Western blot

The detection of EV markers through western blot (WB) was used as a confirmation of the presence of EVs in the solutions obtained at the end of the isolation protocols. The objective was not to perform a detailed characterization of the markers, but to look for differences in their detection between the different methods.

Great inter-method variability has been observed. Briefly, CD63 detection is better in EVs isolated from plasma than in those obtained from serum. Among the plasma-derived EVs, CD133 showed better signal for *centri2500* and *centri13000* than for the *salting out* and the *standard* protocols. The detection of CD133 was also worse in serum-derived sample than in plasma-derived samples. Nonetheless, CD133 detection was better in urine-derived EVs than in the samples obtained from blood (Figure 3B). It is of note that the EVs isolated with *exoquick* could not be used for WB marker analysis using plasma samples due to be impossible to dissolve its pellet.

### Urine

#### EV concentration

In the NTA analysis of the EVs isolated from urine, we have observed that the concentrations were as low as the great majority of the samples have not reached the minimum track-count. Thus, once again the interpretation of NTA data from urine-derived EVs was carried out with caution. *Exoquick* was the method that yields the highest EV concentrations from urine using either NTA or FC, followed by the *standard* protocol. The *centri2500* and *salting out* protocols yielded very similar concentrations. Figures 2E,F summarizes the EV concentration results from the application of the different protocols to urine as measured by NTA and FC, respectively (for *p* values, please see Table S1 in Supplementary Material). In summary, using both NTA and FC, we obtained a similar concentration profile to that of blood but with 10–20 times lower concentrations.

#### EVs size

The modes of the size distributions obtained with the different protocols were similar and, the average of these modes (207 nm) was similar to that achieved in blood samples (228 nm). As it happened with blood samples, the smaller EVs were isolated with the *standard* method (162.5 nm) comparing to the other protocols (Figure 2G). The results of the size assessed by NTA were, once again, consistent with the observations done using EM. In most of these urine samples, the number of contaminating particles that could not be considered EVs was higher than in blood (Figure 3A).

#### RNA concentration in EVs

Higher RNA concentrations were obtained from EVs isolated with the *standard* (33 ng/μl) and *salting out* (25.9 ng/μl) methods and even higher than the concentrations obtained from blood samples, where the EV concentration was from 10 to 20 times higher than in urine (Figure 2H).

#### Western blot

The EV samples isolated from urine using *exoquick*, in contrast to the ones isolated from plasma, can be used for marker analysis with WB (Figure 3B).

In brief, CD133 detection in urine-derived samples is better with precipitant agents (*exoquick* and *salting out* methods) than with centrifugation methods, although the *standard* shows better results in urine and serum than in plasma. On the opposite, CD63 signal is weak in urine-derived samples compared to that of plasma- or serum-derived samples for all methods.

### Correlation with laboratory parameters

We have also tested whether the EV concentration values obtained with different methods and the different types of samples (plasma, serum, or urine) could be reflected in some of the lab parameters measured in blood and urine, especially the ones that are related to the main components of EVs, i.e., lipids and proteins. Furthermore, the analysis of these correlations would serve to test a possible interference in EV quantification produced by these parameters, a phenomenon that has been previously described (31).

Interestingly, a significant correlation has been observed between the concentration, as measured by NTA, of the EVs isolated from plasma with the *centri13000* method and the total cholesterol (*R* = 0.953; *p* = 0.003) and LDL concentrations (*R* = 0.935; *p* = 0.006) in blood. We have also detected a significant correlation between the NTA-measured concentration of the plasma-derived EVs isolated using the *standard* method and the concentration of triglycerides in blood (*R* = 0.789; *p* = 0.007).

No significant correlation has been found between the concentrations of the EVs of specific cell-origins and the concentrations of the respective source cells in blood. The concentrations of CD61+ (platelet origin) or CD45+ (leukocyte origin) EVs are plotted in Figure S1 in Supplementary Material.

In regard to the EVs isolated from urine, the only significant correlation we have observed is the one between the density of urine and concentration of EVs, measured with both NTA and FC, isolated with the *salting out* protocol (*R* = 0.841; *p* = 0.002 for NTA and *R* = 1.000; *p* = > 0.001 for FC).

## Discussion

In the present work, we have studied and compared several widely used methods for the isolation of EVs, including differential centrifugation, agglutination, precipitation, and the one considered the standard that includes ultracentrifugation (plus filter). All methods under study can be applied using relatively simple technology, with the exception of ultracentrifugation, which must be performed with an instrumentation that, even if it is easy to use, is not usually found in most hospital laboratories. The election of one or other method as the most suitable one to be used in a hospital setting greatly depends on the goals to be reached with the method, which could be, among others: to maximize the final EV concentration, to obtain high levels of purity as measured by markers and several classical characteristics of EVs, to select one of the three fundamental types of EVs (exosomes, microvesicles, and apoptotic bodies) or to get the less time and/or money consuming protocol. We have set the first two as preferential aims, leaving the rest out of the scope of this work.

### Isolating EVs

We have observed that, besides being the method that can be implemented most easily (it is quick and relies on very little technology), *exoquick* is also the method that yields, in a statistically significant manner, the highest concentration of EVs (as measured by NTA and FC) compared to the other four isolation protocols. The EV quantity is even higher when using serum as the starting sample. On top of that, to dissolve the pellet obtained using *exoquick* from serum-derived samples is notably easier than plasma-derived samples. Nonetheless, the considerably higher EV concentrations obtained with *exoquick* (23 times higher than those obtained with the *standard* protocol) could be linked to the aggregation and precipitation of other elements in suspension in the sample that are not necessarily EVs; as it can be observed in the images obtained by EM. Taylor and colleagues (32) demonstrated that using *exoquick* more EVs are isolated than using ultracentrifugation (*standard*), chromatography, and magnetic beads, and with a higher purity of exosomal RNA and proteins. Our results only partially coincide with the observations of Taylor and colleagues, as the RNA concentration we obtained with *exoquick* is lower than that yielded by the *standard* method. In another study that compared the *exoquick* method with the *standard* method, the authors concluded that a combination of these two methods is the protocol that yields the highest EV counts, although exosomes of higher quality were obtained combining the *standard* method with the sucrose density gradient (33). Yet, *Exoquick* is the most expensive of the methods used in the present work.

The differences in EV concentration between the two centrifugation methods (*centri2500* and *centri13000*) are not statistically significant. The sole differences are that the cluster of EVs observed by FC in the FSC/SSC dotplot shows less debris around in the case of *centri13000* and that the EVs isolated with this method also show a stronger labeling of CD63 in WB. The conclusions reached at the workshop of the Scientific and Standardization Committee of the International Society of Thrombosis and Hemostasis to promote the use of these protocols (15) and aimed to reducing the variability due to a resuspension of the pellet (24). Nonetheless, in our opinion, the main drawback of this proposal is that, as the EVs are not concentrated in a pellet–like, we performed with the final centrifugation at 20,000 × *g*, a pellet-washing step cannot be introduced and EVs are maintained in dissolution along with many other contaminating particles such as protein aggregates. Regarding size, very similar EV sizes have been obtained with these two methods, even when measuring size on EM imagery. These suggest that the second centrifugation is probably not that critical and could vary, at least between 2,500 and 13,000 × *g*, with the objective of eliminating cell debris. Moreover, these methods collect six times higher EV concentrations than the *standard* protocols, what can be explained by the fact that they are less restrictive methods. Finally, the technical requirements for the use of these methods are usually met in most basic research laboratories and they are considerably less time-consuming than the *standard* method.

Ultracentrifugation is nowadays the “*gold standard*” method for the isolation of EVs, fundamentally exosomes. With the aim of finding alternative methods to this protocol, Brownlee and colleagues (25) have recently described a new method called *salting out*, based on the precipitation of EVs using the aggregate of acetic acid. In the present study, the *salting out* method yielded the lowest EV concentrations when compared to the other protocols, although showing similar values to those obtained with the *standard* method as the authors of the aforementioned article also pointed. It has to be noted, though, that Brownlee and colleagues isolated EVs from cell culture supernatants and, thus, comparisons with the present work must be done with caution.

Although out of the main objectives of this work, we have also compared the EVs isolated from three different types of samples: plasma, serum, and urine. We have observed that higher concentrations of EVs are obtained from serum than from plasma for all methods, and 10–20 times more, depending on the method, when comparing plasma with urine. As comparisons between serum and plasma have been performed by other authors (34, 35), we just present our results.

The EV size distributions that we have obtained with the *exoquick*, *salting out*, *centri2500*, and *centri13000* methods are very similar, being the EVs with a size below 200 nm the most abundant. Nevertheless, a cluster of EVs can be observed with a size around 500–600 nm that could represent the population of microparticles. On the contrary, the standard method isolates smaller EVs as it uses a 0.22 μm-pore filter leaving out the bigger EVs (microparticles and apoptotic bodies). We agree with Jy and colleagues (36) that the capacity of the first four methods to isolate the bigger EVs can be useful when applying these protocols in clinical practice.

In the case of urine, very low EV concentrations have been obtained with the five methods and, thus, we recommend not to dilute or to dilute very little urine-derived samples before analyzing them by NTA, FC, and WB. Once again, *exoquick* was the method that yields the highest concentrations according to other authors’ results (37). Certainly, when using urine samples, it would be of great consequence to avoid contaminating proteins such as Tamm–Horsfall, which traps EVs but it can be removed with the simple addition of dithiothreitol and heat (38). Furthermore, Rood et al. (18) suggest that the most effective method in terms of purity for urine-derived EVs to undergo downstream proteomic analysis is the combination of ultracentrifugation followed by size-exclusion chromatography. The major disadvantage of this protocol would be that it is time-consuming and it requires of specific infrastructure that make it difficult to be compatible with clinical applications.

### Detecting and characterizing EVs

During the processing, after the centrifugation at 20,000 × *g* for 20 min, a fine lipidic layer could be observed in some of the samples. This corresponded to a FC image with a higher EV density (data not shown). Nevertheless, the presence of this layer did not show correlation with NTA results. It is well known that the density and size of the EVs can overlap with these of lipoproteins and this can produce artifactual results in FC analyses (31). Besides, we have found positive correlation between the LDL levels in blood and the concentration of EVs obtained with several methods, which suggests that, when isolating the EVs, some LDL particles are also dragged and counted as EVs. One approach to measure the purity of EVs is the EV/protein ratio (39), a method that is easy to use and yield reproducible results. However, it remains out of the scope of the present work.

The most widely used methods for the quantification of EVs are NTA and FC. According to our data, the results yielded by these two methods are not interchangeable, probably because the size ranges that they can analyze are different. The correlation between the two methods would be better studied using only the concentration of EVs larger than 400 nm, as this is the minimum size for the FC analysis. Nevertheless, we have looked for correlation using concentration results for EVs larger than 400 nm in another dataset (data not shown) and we have found none. Thus, we consider that these two quantification methods do not exclude each other but are complementary, as NTA gives more accurate counts but FC allows the characterization of distinct cellular origins.

From the comparison of the methods that we have used to study the size of EVs, we can conclude that, while the NTA, as it allows to recover information from individual particles, allows to obtain and compare size distributions, EM provides more robust information on the characteristics of EVs but size distributions cannot be obtained through EM imagery. Furthermore, NTA has the advantage of performing a multiple analysis in few minutes.

Tetraspanins have been widely used as general markers of EVs; however, during the last years, some works have provided evidence that not all vesicles express them at the same levels suggesting that different EV subsets could coexist in the same pellet (40, 41). In the case of urine-derived EVs, our results present low or undetectable levels of CD63 except for those obtained with the *standard* protocol (Figure 3B). Both the previously described lack of CD63 in urine-derived EVs larger than 100 nm (42) and its expression in EVs obtained with the *standard* protocol (43, 44) are congruent with our results. In the other hand, we found expression of CD133 with all the methods. In agreement with other authors, we concluded that the presence of CD133+/CD63− EVs demonstrate the recovery of the large ones that usually express this pattern of markers (45–47). Moreover, Bobrie and colleagues described the CD63 as a variable marker found only in a fraction of the sucrose gradient (40), which implies questioning the use of CD63 as a standard EV marker (48). Finally, the expression of CD63 is susceptible to SCORT regulation leading to the blockage of the budding of this EV subset (49) and this mechanism could hypothetically be more frequent in urine-derived EVs. Regarding to plasma-derived EVs, the detection of the opposite pattern (CD133−/CD63+) in the EVs obtained with *standard* protocol unravel the isolation of a specific EV fraction, being probably only exosomes (46).

Regarding the RNA concentrations yielded by the different EV isolation methods, we have observed great variability. Although Taylor and colleagues conclude that *exoquick* isolates more than, among other methods, ultracentrifugation (32), we have observed, unexpectedly, that the RNA concentrations obtained with the different methods are very similar, despite the notable differences in EV concentrations. Surprisingly, high RNA concentrations were obtained from urine (especially when using the *salting out* and *standard* methods), concentrations similar to or even higher than those obtained from plasma and serum, regardless of EV concentrations being between 10 and 20 times lower. These results lead us to think that, as we have not used RNAses, we are measuring the concentration not only of the RNA contained in the EVs but of the free RNA. In a position paper of the International Society of Extracellular Vesicles, the authors suggest that the use of RNAses only removes the free RNA not specifically bound to EVs, while their use in combination with proteases also removes the nucleoproteic complexes (50). In any case, if the final objective is to use the RNA as a source of potential biomarker, we believe that it would be useful to preserve not only the RNA inside the EVs but the RNA stuck to their membrane.

### EVs from bench to bedside

The importance of the study of EVs in a hospital setting to complement the diagnosis and prognosis of several diseases has been well demonstrated (51–53). Moreover, their application in therapeutic approaches has already been tested in clinical trials with promising results (54). Nonetheless, we believe that the workflows from the collection of the samples aimed at the isolation, processing, and characterization of EVs to yield significant results to be applied on patients need to be urgently standardized. Specifically, the different isolation method can yield different types of EVs and, thus, omics studies performed on them could give incomparable results. Besides, not all methods are applicable in a hospital setting.

**Figure 4:**
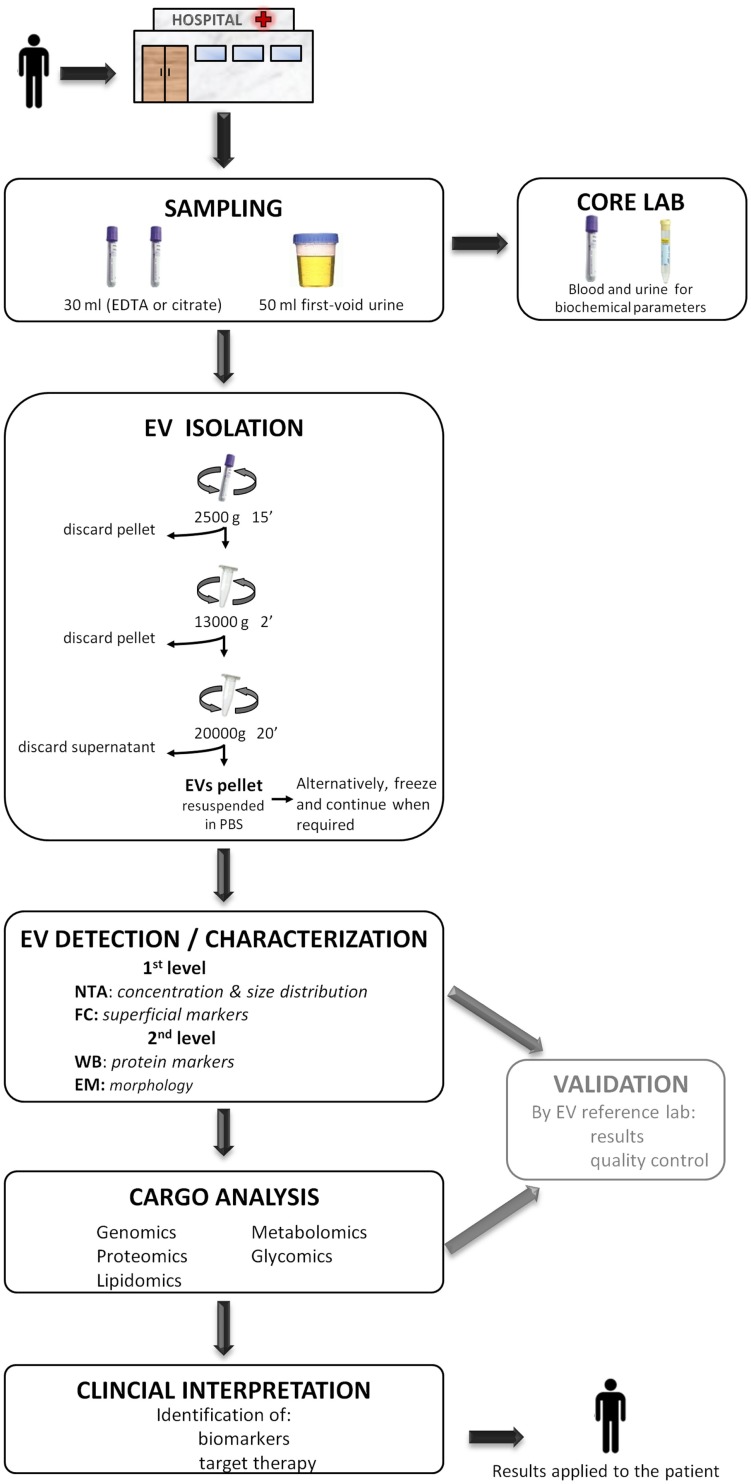
**A proposed workflow for the study of extracellular vesicles (EVs) in a hospital setting**. Patients visited during the morning in the hospital, preferentially on fasting, undergo sample collection of 30 ml of blood (EDTA or citrate) and 50 ml of the first void urine. Immediately, 15 ml of blood and 40 ml of urine are destined to the EV isolation protocol to obtain a pellet and the rest 15 and 10 ml are sent to the core laboratory to analyze biochemical parameters. The obtained EV pellet resuspended in PBS could optionally be frozen at −80°C and continue when required. Next, the detection/characterization of EV is divided in two levels for quantification, size [nanoparticle tracking analysis (NTA)], and initial characterization with flow cytometry (FC) followed by an extensive description with western blot (WB) and electronic microscopy (EM). Subsequently, the analysis of EV cargo with several omics platforms allows the identification of specific compounds carried by EVs. EVs detection and their cargo analysis could optionally be referenced, at least during the initial setting of this workflow, to an expert EV laboratory in order to provide a validation of the results and pass a quality control test. Finally, the detected molecules are interpreted in the whole context of the patient with the aim of identifying biomarkers or a target for a putative therapy. The results provided by the study of EV are applied back to the patient improving the diagnosis or course of the disease.

With aim of contributing to this debate and in accordance to the results of the present work, we consider that the *centri13000* method is the most suitable one to be used in a hospital setting as (a) it requires a simple infrastructure (and does not require ultracentrifuge) that is available in any general laboratory, (b) isolates EVs with similar characteristics to the ones isolated with the *standard* method but in higher concentrations, (c) it recovers not only small EVs as standard does but also the largest, and (d) in analysis with FC and WB showed less contamination when comparing with *centri2500*. We concur with Deun and colleagues (55) in that it is necessary for a validation of the isolation procedure and we propose that this validation could be carried out in referent laboratories lead by group with great expertise in the study of EVs. The results obtained in the hospital setting should be compared to those obtained by the reference lab to assure a quality control. On the other, for the posterior detection and characterization of EVs, we recommend to analyze them with at least one quantification method (NTA or FC) and one characterization method (WB, EM, or FC) as they provide complementary information. Figure 4 summarizes a proposed workflow based on the discussion above.

To conclude, the isolation of EVs, at least for plasma-derived ones, through differential centrifugation at medium speed (*centri13000*) and their posterior analysis with at least one quantification method (NTA, for example) and another characterization method (FC or WB, for example) could fit in a workflow that goes from the patient to lab and all the way back to the patient and would contribute to face several health problems.

## Conflict of Interest Statement

The authors declare that the research was conducted in the absence of any commercial or financial relationships that could be construed as a potential conflict of interest.

## Supplementary Material

The Supplementary Material for this article can be found online at http://www.frontiersin.org/Jounal/10.3389/fimmu.2015.00050/abstract
