# Supplementary figures and images for: Methods for Extracellular Vesicles Isolation in a Hospital Setting
Source: Front Immunol. 2015 Feb 13;6:50. doi: 10.3389/fimmu.2015.00050 (PMC4327731; doi:10.3389/fimmu.2015.00050)

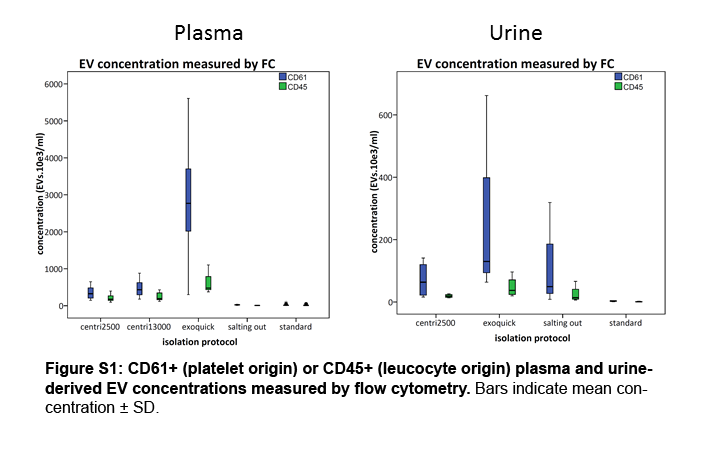

Supplement: Supplementary file 2 [file Image_1.TIF]
